# Demonstration of Adaptive Functional Differences Seen in Kidneys Accompanying a Nonfunctioning/Hypofunctioning Partner, using Camera Based Tc 99m MAG3 Clearance Measurement Technique

**DOI:** 10.4274/Mirt.309

**Published:** 2012-08-01

**Authors:** Burcu Esen Akkaş, Gülin Uçmak Vural, Ümit Özgür Akdemir, Neşe İlgin Karabacak

**Affiliations:** 1 Ankara Oncology Research and Training Hospital, Department of Nuclear Medicine, Ankara, Turkey; 2 Gazi University Medical Faculty, Department of Nuclear Medicine, Ankara Turkey

**Keywords:** Kidney function tests, 99mTc MAG3, radioisotope renography

## Abstract

**Objective:** The aim of this study was to demonstrate the functional compensation that occurs in kidneys which accompany a partner with total or partial loss of renal functioning mass, using camera-based Tc 99m MAG3 clearance technique.

**Material and Methods:** Eighty five patients (43M, 42F, age: 44.8±12.6, range: 18-77 years) with normal serum creatinine levels and normal (<Grade 1) Tc 99m MAG3 renogram curves were enrolled for this retrospective study. Patients were grouped as having; group 1: solitary normal kidney (unilateral atrophied/agenetic) (n=23), group 2: normal kidney with contralateral hypoplasic/hypofunctioning kidney (split renal function<30%), (n=24), group 3: bilateral normal kidneys (n=38). The measured camera based Tc 99m MAG3 clearances of normal kidneys in each group were compared.

**Results:** Total Tc 99m MAG3 clearances (mL/min/1.73m2) were significantly lower in group 1 and group 2 compared to group 3 (281.5±46, 260.5±61.7 and 316.1±84, respectively). Highest isolated Tc 99m MAG3 clearances among normal functioning kidneys were observed in group 1 (281.5±45.6) followed by group 2 (204.4±55) and group 3 (157.5±44). Moderate negative correlation was detected between the Tc99m MAG3 clearances of normal kidneys and contralateral renal function (r=-0.5, p<0.001).

**Conclusion:** Normal kidneys can compensate for the loss of contralateral kidney function via increasing their clearances, which seems to be dependent on the residual function of their partner. Camera based Tc 99m MAG3 clearance measurement is an objective method to demonstrate compensatory differences in renal function seen between kidneys with contralateral normofunctioning, hypofunctioning and nonfunctioning partner.

**Conflict of interest:**None declared.

## INTRODUCTION

Compensatory renal hypertrophy (CRH) is a multistep adaptive process and is known to occur in the remaining kidney after the partial or total loss of renal mass. It was reported that the remaining kidney began increasing in size to compensate the missing partner within several days after unilateral nephrectomy (1,2,3). More than a 48% increase in renal volume was reported in rats after unilateral nephrectomy (1). Enlargement of nephrons occurs in response to subtotal renal ablation or to patchy loss of nephrons due to renal diseases (4). The changes related to growth are paralleled by significant increments in proximal convoluted tubules, distal convoluted tubules and the cortical collecting ducts (1). In adults, compensatory growth of the remaining kidney occurs mainly through the hypertrophy of tubular cells (5).

Technetium-99m mercaptoacetyltriglycine (Tc 99m MAG3) has been used to determine the tubular functions of kidneys in adults and children for over 20 years. Tc 99m MAG3 is superior to many other renal agents and improves the quantification of renal clearance by virtue of its good imaging properties and low radiation exposure (6). The clearance of Tc 99m MAG3 is proportional to the effective renal plasma flow (orthoioidohippurate (OIH) clearance) and can be used as an index of renal function. The camera based Tc 99m MAG3 clearance measurement is a validated method and is accepted to provide a more reliable measure of renal function than the serum creatinine levels or the creatinine clearance method (7,8). Bubeck et al. described the Tc 99m MAG3 clearance as the tubular extraction rate of kidneys corresponding to the “virtual plasma volume per minute from which a substance is completely removed by tubular extraction” (9). 

Current literature supports the use of Tc 99m MAG3 clearance to estimate the renal functions in renal transplant donors and patients undergoing unilateral nephrectomy (10,11). Predicting the compensational capacity of the remaining kidney following the loss of its partner by a non-invasive test is important to avoid post operative renal insufficiency. 

Combining the physiological aspects of CRH with the use of Tc 99m MAG3 clearance as an indicator of tubular renal functions, we designed this study to further evaluate whether the camera-based Tc 99m MAG3 clearance technique can demonstrate the functional compensation that occur in normal functioning kidneys with contralateral nonfunctioning or hypofunctioning kidney in patients with normal serum creatinine levels.

## MATERIALS AND METHODS

**Patients**

The subjects (n=85, 43 male, 42 female patients, mean age: 44.8±12.6, age range: 18-77) were selected retrospectively among a group referred to the nuclear medicine department as prospective donors for transplantation, patients with a recent upper urinary tract infection and for the evaluation of renal functions accompanying a contralateral aplasic or hypoplasic kidney. Patients were grouped as; group 1: patients with solitary normal kidney (unilateral atrophied or agenetic) (n: 23, 9 male, 14 female, mean age: 45.4±12.3), group 2: patients with a normal kidney and contralateral hypofunctioning kidney (split renal function <30%), (n: 24, 13 male, 11 female, mean age: 46.8±11.8), group 3: patients with bilateral normal kidneys (n: 38, 21 male, 17 female, mean age: 43.3±13.4). Age and gender did not differ between the groups. 

Each subject was required to fit the following inclusion criteria; normal serum creatinine levels (0.7-1.2 mg/dl), no morphological abnormalities in ultrasound and a normal (<Grade 1) renogram for one kidney following a dynamic renal scan with Tc 99m MAG3 (12). 

Patients with renal and collective duct abnormalities (such as congenital renal malformations, ectopic kidneys, and duplex collecting system anomalies) were not included in the study group. We did not measure creatinine clearances in this study since the MAG3 clearance measurement is known to be a more reliable criterion for the changes in renal functions than the creatinine clearance measurements (7,8,13,14,15) and in order to avoid patient inconvenience by collecting 24 hours urine samples.

**Data Acquisition and Analysis**

The subjects were hydrated with approximately 500 mLs of water 30 minutes before the study. Images were acquired in a 128?128 matrix with a gamma camera equipped with a low-energy all-purpose collimator (Millenium MG, General Electric, Milwaukee, WI, USA). Each subject was imaged supine with the kidneys and bladder in the field of view. After the i.v. injection of 5 mCi (185 MBq) of Tc 99m MAG3 (TechneScan MAG3, Mallinckrodt Medical Inc, Maryland Heights, MO, USA), serial 2-seconds-per-frame digital images were obtained for the first 48 seconds followed by 15 and 30-seconds-per-frame images for a total study duration of 40 minutes. At the end of the acquisition, additional a post-void 2-minute static image was obtained with the patient in the same supine position. 

The data were processed using the QuantEM 2.0 software (developed at Emory University and licensed by Emory University to GE Healthcare), which was developed specifically for Tc 99m MAG3 renography (8,16). The software determines the percent injected dose in each kidney from 1-2.5 minutes postinjection and uses an algorithm derived from a multicenter trial to convert the sum of the percent injected dose in the left and right kidneys to a global MAG3 clearance (mL/min/1.73 m^2^) (8). The pre-injection and post-injection syringes containing the 5 mCi (185MBq) dose were counted on the camera; counts in the post-injection syringe were corrected for decay and subtracted from the counts in the pre-injection syringe to determine counts injected. To obtain percent injected dose in each kidney, the kidney counts from 1–2.5 minutes post-injection were corrected for background, infiltration, attenuation and renal depth and divided by the counts injected (8). Renal cortical region of interests (ROI) both for renal cortex and whole kidney were drawn manually by the same physician, and renogram curves were generated semi automatically by QuantEM 2.0 software. The camera based Tc 99m MAG3 clearances were calculated using the following formula (17):

([(TAF) (LKc – BG)/ e -0.137(x-1.1)] + [(TAF) (RKc-BG))/ e -0.137(x-1.1)])

Counts injected

Tc 99m MAG3 clearance = 10.8 (% injected dose in the kidney) (BSA /1.73 m^2^) – 2.5The percent injected dose in the kidneys for time interval between 1-2.5 min postinjection was determined using the following equation (17):

BSA: body surface area, TAF: table attenuation factor, x: renal depth, 0.137: effective attenuation coefficient of 99mTc-MIBin tissue, RKc: right kidney counts, LKc: left kidney counts, BG: background counts

**Statistical Analysis**

The total Tc 99m MAG3 clearances of the groups and the Tc 99m MAG3 clearances of normal functioning kidneys of each patient group were compared by the Kruskal Wallis test. The statistical difference was considered as significant when the p value was <0.05. The correlation between the isolated Tc 99m MAG3 clearances of normal kidneys and the contralateral split renal function was analyzed using Spearman’s correlation test.

## RESULTS

Tc 99m MAG3 curve parameters, renal functions for all kidneys and serum creatinine levels are presented in Table 1.

All patients in the study population had normal serum creatinine levels. The average of creatinine levels were 0.9±0.09 mg/dL in group 1, 1±0.08 mg/dL in group 2 and 0.8±0.09 mg/dL in group 3.

Split renal functions were 99.9% and 0.1% for kidneys of group 1 patients, 80.2% and 19.8% for kidneys of group 2 patients and 51.7% and 49.3% for kidneys of group 3 patients.

The mean of total Tc 99m MAG3 clearance in patients with bilateral normal kidneys (group 3) was 316.1±84.4 mL/min/1.73 m^2^ in our study. The mean of total Tc 99m MAG3 clearance values measured in other groups were 281.5±45.6 mL/min/1.73 m^2^ in patients with a solitary normal kidney (group 1) and 260.5±61.7 mL/min/1.73 m^2^ in patients with a contralateral hypofunctioning kidney (group 2). The differences between the total 99mTc-MAG3 clearance values of each group were statistically significant (p=0.01). We observed statistically significant decrease in the total Tc 99m MAG3 clearance measurements of these two groups when compared to patients who have bilateral normal kidneys.

**Figure 1:**
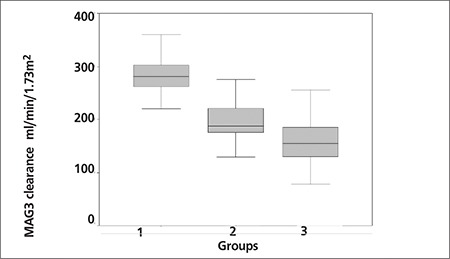
The graphic demonstrates the Tc 99m MAG3 clearance measurements of normal functioning kidneys in each patient group. The compensational increase in Tc 99m MAG3 clearance of normal functioning kidneys due to contralateral kidney mass and the differences of Tc 99m MAG3 clearances among groups are clearly seen

Although the total Tc 99m MAG3 clearances were lower in group 1 and group 2 than group 3, the highest Tc 99m MAG3 clearance among normal kidneys of each group was observed in group 1 (281.5±45.6 mL/min/1.73 m^2^) which is followed by group 2 (204.4±55 mL/min/1.73 m^2^) and then group 3 (157.5±44 mL/min/1.73 m^2^, the average of Tc 99m MAG3 clearance measurements of left and right kidney), p=0.0001. In other words, we observed that solitary normal kidneys had significantly higher Tc 99m MAG3 clearance values compared to kidneys accompanying either a hypofunctioning or a normal functioning kidney (Figure 1). Similarly, kidneys accompanying a hypofunctioning kidney have higher Tc 99m MAG3 clearance values compared to kidneys accompanying a normal functioning kidney (p=0.002) (Figure 1). Additionally, a moderate negative correlation was detected between the Tc 99m MAG3 clearances of normal kidneys and the contralateral split renal function (r=-0.5, p=0.0001). 

## DISCUSSION

Compensatory renal growth occurs after any pathology that results in the loss of contralateral kidney mass and function. This is an adaptive process which results in an increase of size and the functional capacity of the remaining kidney. The mechanisms of renal hypertrophy in response to loss of renal mass are fundamental to understand and monitor the changes in renal functions. Studies in human fetuses with either agenesic or contralateral dysplastic kidney showed that the size of the solitary functional kidney was larger than normal suggesting that a compensatory renal growth had occurred (18,19). As nephrogenesis is complete before birth in human, enlargement of the remaining kidney cannot involve the formation of new nephrons (2). The functional adaptation to the loss of one kidney consists mostly of an increase in the glomerular filtration rate of the remaining kidney, and hypertrophy of nephron parts, mainly of the proximal tubular cells (5). Evidence demonstrated that immediately after the removal of one kidney, the remaining kidney was subject to hyperfiltration (4,20).

Tubular cell hypertrophy was reported to occur in several pathologies that affect the kidneys (5). Tubular cell hypertrophy seen after the partial or total loss of contralateral kidney function occurs through a process involving cell cycle-dependent mechanisms (5,21). Mesangial cell proliferation initiated by unilateral nephrectomy was reported in previous series and it was shown that these cells induced tubular hypertrophy by secreting growth factors (5). Transforming growth factor-β (TGF-β) is considered to be a pivotal factor in the progression of compensatory renal growth of the remaining kidney (22). On the basis of these histological data, in this study, we aimed to demonstrate the functional differences seen in normal functioning kidneys with renal partners having diminished functions by monitoring renal tubular functions. 

Tc 99m MAG3 has high extraction efficiency from blood to functional kidneys followed by the active excretion by the tubular system. It has become the radiopharmaceutical of choice for its higher renal extraction compared with 99mTc-MIBdiethylenetriaminepentaacetic acid (DTPA), better imaging characteristics and lower radiation exposure to patients. Additionally, the clearance of Tc 99m MAG3 is closely correlated with orthoioidohippurate clearance (effective renal plasma flow), and can be used as an index of renal function (23,24). Tc 99m MAG3 clearance can be measured by the conventional single-injection multi-sample plasma clearance methods, by any of the several simplified methods based on a single timed plasma sample or by the camera based clearance methods, as well. 

The camera-based Tc 99m MAG3 clearance is an easily performed, reproducible, objective and a validated method that can be obtained at the time of scanning without the need to provide blood or urine sampling (8,17). In the present study, we found that mean total Tc 99m MAG3 clearance in patients with bilateral normal kidneys was 316.1±84.4 mL/min/1.73 m^2^. These results were in parallel with the plasma sample Tc 99m MAG3 clearance values reported in SNM guidelines and with the mean values measured in two separate populations of potential renal donors at different institutions which were 304±70 and 317±74 mL/min/1.73 m^2^ (25,26). 

The camera-based MAG3 clearance method has greater sensitivity to changes in renal function than the creatinine clearance measurement and therefore superior for monitoring changes in renal function (7,8,13,14,15).

It is proved that Tc 99m MAG3 clearance measurement provides important information to estimate the postoperative renal functions in renal transplant donors and patients undergoing unilateral nephrectomy (10,11). Authors reported that preoperative MAG3 clearance of the remaining kidney significantly correlate with postoperative creatinine clearance; such that, preoperative MAG3 clearance of the remaining kidney<130 ml/min/1.73 m^2^ is indicative of postoperative renal insufficiency (10). Predicting the compensational capacity of the remaining kidney following the loss of its partner by a non-invasive test is important to avoid post operative renal insufficiency. 

**Figure 2:**
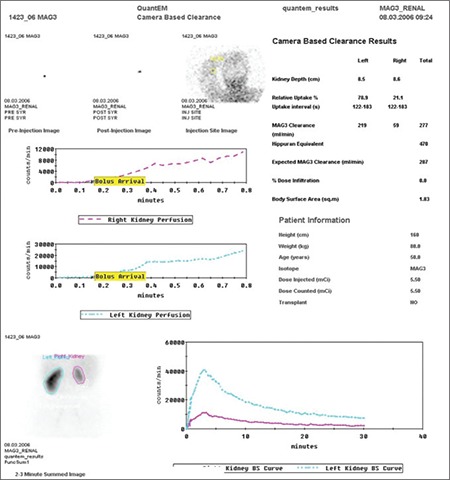
The standard display of QuantEM review screen shows the demographic data, the injected dose, the dose counted on camera, dose infiltration, relative renal uptakes, Tc 99m MAG3 clearance for each kidney,total Tc 99m MAG3 clearance and hippuran equivalent values on the right panel. Kidney perfusion and function curves are demonstrated in the left panel and the lower panel. This review screen belongs to a patient from group2. The Tc 99m MAG3 clearances of the normal functioning kidney and the hypofunctioning kidney with a split renal function of 21% were measured as 219 mL/min and 59 mL/min, respectively

Camera based Tc 99m MAG3 clearance programs are available on most nuclear medicine camera systems. The particular software program, QuantEM used for this study, is currently available on the General Electric computer systems (Figure 2). Measurement of the Tc 99m MAG3 clearance at the time of scanning adds important information to help direct patient management and to detect early loss of kidney function even before predominant changes occur in creatinine clearance values or in renogram curves (8,10,11,16). From another point of view, as an objective index of renal tubular function, the Tc 99m MAG3 clearance measurements may serve as an indicator for compensatory renal changes that are reported to occur mainly in renal tubular systems. 

However, the present study has some limitations that mainly arise from the methodology of camera-based clearance methods. Even though camera-based techniques are more popular than plasma sample techniques because they avoid the necessity of delayed plasma sample(s), they are dependent on an accurate estimation of renal depth to correct for soft-tissue attenuation (27). In this study, renal depth is estimated from the regression formula, based on height and weight, which is another source of bias. Errors in absolute and relative function measurements can be introduced when the kidneys are assumed to lie at the same depth but these differences may be interpreted as differences in renal functions. However, authors reported that the average absolute difference in renal depth was 6.1 mm and 84% of patients have a difference of less than 1 cm (27).

In this study, we observed that Tc 99m MAG3 clearance values were significantly increased in solitary normal kidneys compared to kidneys accompanying either a normal functioning or a hypofunctioning partner. The Tc 99m MAG3 clearance of normal functioning kidney was inversely correlated to the contralateral functioning kidney mass. When the contralateral kidney function was less than normal, the accompanying normal functioning kidney had higher Tc 99m MAG3 clearance values compared to each of bilateral normal functioning kidneys, but lesser Tc 99m MAG3 clearance than solitary normal functioning kidneys. In this study, we observed a negative moderate correlation between clearance values of normal functioning kidneys and contralateral split renal functions. We considered that the reason for a moderate correlation may be originated from the patient selection criteria, such that; we selected 3 group of patients with the hypofunctioning kidney’s split renal function of <1% (group 1), <30% (group 2) and 46-54% (group 3). The split renal functions were homogeneous within each group with no intra-group variability. We considered that further research studies dealing with larger group of patients having split renal functions that vary within a large spectrum may be warranted to test whether a strong correlation is present between the normal kidney clearance and the contralateral diminished renal function. 

We observed that among normal functioning kidneys of each group, the highest clearance values were achieved by solitary normal functioning kidneys. Even in some of the patients, the Tc 99m MAG3 clearance of the solitary kidney was comparable to the total clearance values of bilateral normal kidneys. We considered these findings significant as an indicator of compensational processes due the loss of the contralateral kidney’s function and these processes seem to be dependent on the function of abnormal kidney. 

Even though the parameters calculated from gamma camera renography using Tc 99m MAG3 represent split renal functions and are not specified to compensate renal functions, we considered that the only possible mechanism responsible from these objective findings is the compensational functional renal changes occur in the accompanying normal functioning kidneys. However, further research studies performed prospectively on the same patient population before and after renal damage, are warranted to prove our observations.

In conclusion, the difference that we observed between the individual clearance values of each normal functioning kidney provided some confirmation of the adaptive changes where the remaining kidney shows some adaptation and growth response to keep the glomerular filtration rate and renal tubular functions in normal ranges following unilateral partial or total nephrectomy. In cases where a normal kidney accompanies a hypofunctioning kidney, in order to maintain homeostasis, functional compensation occurs depending on the contralateral kidney mass and results in an increase in Tc 99m MAG3 clearance. The camera based Tc 99m MAG3 clearance measurement technique can demonstrate the functional differences, suggestive of compensatory changes, between normal functioning kidneys with contralateral normofunctioning, hypofunctioning and nonfunctioning kidneys. 

## Figures and Tables

**Table 1:**
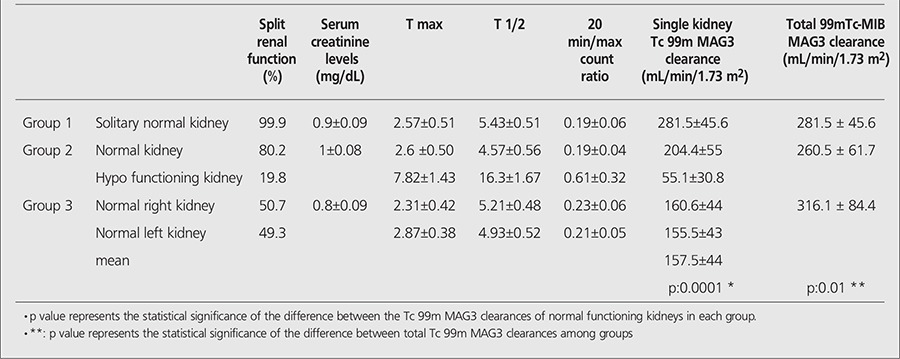
Table 1. Renogram parameters and serum creatinine levels of all patients in the study group
